# Research on PF-SLAM Indoor Pedestrian Localization Algorithm Based on Feature Point Map

**DOI:** 10.3390/mi9060267

**Published:** 2018-05-28

**Authors:** Jingjing Shi, Mingrong Ren, Pu Wang, Juan Meng

**Affiliations:** 1 College of Automation, Faculty of Information Technology, Beijing University of Technology, Beijing 100124, China; shijingjing123@emails.bjut.edu.cn (J.S.); wangpu@bjut.edu.cn (P.W.); S201761125@emails.bjut.edu.cn (J.M.); 2 Engineering Research Center of Digital Community, Ministry of Education, Beijing 100124, China; 3 Beijing Key Laboratory of Computational Intelligence and Intelligent Systems, Beijing 100124, China

**Keywords:** indoor localization, inertial navigation system (INS), simultaneous localization and map building (SLAM) algorithm, particle filtering, feature point matching

## Abstract

Recently, the map matching-assisted positioning method based on micro-electromechanical systems (MEMS) inertial devices has become a research hotspot for indoor pedestrian positioning; however, these are based on existing indoor electronic maps. In this paper, without prior knowledge of the map and through building an indoor main path feature point map combined with the simultaneous localization and map building (SLAM) particle filter (PF-SLAM) algorithm idea, a PF-SLAM indoor pedestrian location algorithm based on a feature point map was proposed through the inertial measurement unit to improve indoor pedestrian positioning accuracy. Aiming at the problem of inaccurate heading angle estimation in the pedestrian dead reckoning (PDR) algorithm, a turn-straight-state threshold detection method was proposed that corrected the difference of the heading angles during the straight-line walking of pedestrians to suppress the error accumulation of the heading angle. Aiming at the particles that are severely divergent at the corners, a feature point matching algorithm was proposed to correct the pedestrian position error. Furthermore, the turning point extracted the main path that failed to match the current feature point map as a new feature point was added to update the map. Through the mutual modification of SLAM and an inertial navigation system (INS) the long-time, high-precision, and low-cost positioning functions of indoor pedestrians were realized.

## 1. Introduction

With the rapid development of modern digital information and the rise of smart cities, location based service (LBS) has attracted increasing attention [1]. From daily outdoor positioning and navigation development to complex indoor environments, the demand for pedestrian indoor positioning and navigation systems is high, for example, the guidance of shopping malls or hospitals, the location index of airports or parking lots, and safe rescue and other professional needs fields [2]. Typical indoor pedestrian positioning technologies mainly include indoor positioning based on wireless networks [3,4], inertial navigation system (INS) based pedestrian dead reckoning (PDR) [5,6,7], positioning based on radio frequency (RF) signals [8,9], positioning technology based on geomagnetic matching [10], and various combinations of positioning technologies.

Among them, the INS solves the difficulty of global positioning system (GPS) in indoor positioning and can perform indoor positioning systematically and independently without relying on an external source. The basic working principle of this system is based on Newton’s classical mechanics law, whereby it measures the acceleration information of the motion carrier in the inertial reference coordinate system, integrates its information with time, and then converts it into the navigation coordinate system through quaternion and other coordinate transformation methods where the velocity information, yaw angle information, and position information in the navigation coordinate system can be obtained [11]. The advantage is that the user can position and navigate by wearing a wearable sensor and, given the small size of these sensors, there is no problem of inconvenient carrying. However, since the navigation information is generated by time integration, and as the sensor itself creates measurement errors, the positioning errors will increase with time, leading to poor long-term positioning accuracy. Especially given the errors of gyroscopes, there are no reliable observations, causing the heading errors to accumulate and positioning failure. How to suppress the accumulation of heading errors is a problem that needs to be solved urgently based on INS’s pedestrian dead reckoning system. In [12], the accumulation error was eliminated by the heading information before and after passing through the same site twice; the experimenter must pass through the same site twice in a test, so there are also errors in the detection of the same location.

The feature point detection assisted map matching algorithm has been embraced by researchers. Most of the map matching algorithms based on feature point detection are based on mastering prior knowledge of map information [13], that is, by selecting points with more obvious features from the building plane information map as feature points [14]. The feature points serve as a reference to correct the positioning error. However, in cases of emergency rescue or indoor maps that are difficult to obtain such as fire rescue, military rescue, and so on, pedestrian positioning based on indoor maps cannot be effectively implemented. The simultaneous localization and map building (SLAM) algorithm originating from a robot does not need to prepare the environment map information in advance and can achieve the function of synchronous positioning and composition [15]. This algorithm has the advantage of relatively simple external sensors that obtain environmental information and does not require a priori map information.

The SLAM algorithm based on particle filtering is referred to as PF-SLAM [16]. As a non-linear filtering algorithm based on Bayesian estimation, the particle filtering method has unique advantages in dealing with the parameter estimation and state filtering problems of non-Gaussian non-linear time-varying systems, which makes the particles eventually converge to the real point of posterior probability. We integrate the observation to formulate the likelihood function, which effectively updates the weights of particles. Thus, the particle effectiveness is enhanced to avoid the “particle degeneracy” problem and improve localization accuracy [17]. At the same time, the particle filter method has been successfully and widely used in the creation of concurrent maps for robot positioning and the indoor pedestrian positioning system based on map matching. The particle filter algorithm has become the most important and effective method for simultaneous positioning and map construction of mobile robots [18].

In the absence of prior knowledge of indoor maps, this paper proposes a PF-SLAM indoor pedestrian location algorithm based on feature point maps by extracting feature points to construct the feature point map, and proposes a turn-straight-state threshold detection method to correct the heading angle difference and to suppress the heading error accumulation. In combination with the SLAM idea, through the feature point matching algorithm, the problem of particle divergence at the turnings due to the inaccuracy of the heading angle error model was solved. Finally, the re-sampling algorithm prevented particles from being exhausted, resulting in inconsistent calculation results of the algorithm, thereby improving the accuracy of indoor pedestrian positioning. The system description is introduced in Section 2. Section 3 gives the details of the feature point map. The proposed PF-SLAM algorithm, presented in Section 4, is tested in real scenarios and the results are summarized in Section 5.

## 2. System Description

**Figure 1:**
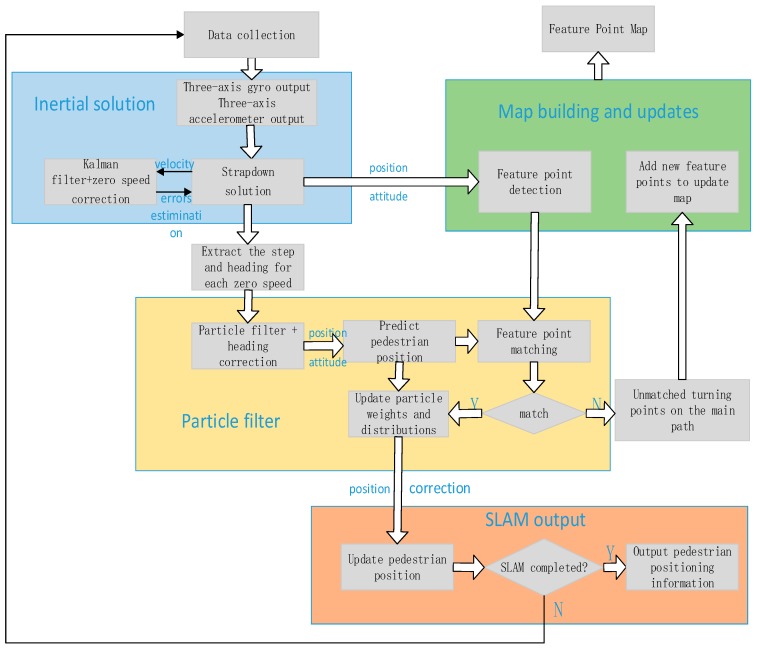
System architecture.

The indoor pedestrian positioning system proposed in this paper is mainly divided into four parts that are based on the following: the inertial navigation algorithm of the Kalman filter and zero velocity update (ZUPT) algorithm, map construction and update, and particle filter to predict pedestrian position, SLAM corrects the pedestrian positioning information and output. Figure 1 shows the system architecture. Inertial sensor devices such as three-axis gyros and accelerometers in the inertial measurement unit (IMU) transmit the sampled data to the computer for inertial system strapdown calculations, a Kalman filter is introduced into ZUPT techniques [19] to suppress inertial device errors. On the basis of the corrected position and attitude of the output, feature point detection is introduced to construct the original feature point map. At the same time, we extracted the step length and heading for each zero speed, using a particle filter algorithm based on pedestrian dead reckoning to predict the next position of the pedestrian. In the particle filter algorithm, a turn-straight-state threshold detection method was introduced to correct the heading in the pedestrian’s straight-line walking phase, suppress the heading cumulative error, and predict the heading and posture of the pedestrian after correction through the extracted turning points of the pedestrian in order to perform feature point matching. If it matches the existing feature point database, it updates the particle weight and particle distribution to update the pedestrian position; if it cannot match the existing feature point database, the unmatched turning points on the main path will be added to the feature point map as a new feature point to expand and update the map. Pedestrian poses are corrected through feature point matching; at the same time, the corrected pedestrian poses are used to supplement and update the feature point map in order to obtain more accurate pedestrian poses and feature point map information after correction.

## 3. Feature Point Map

### 3.1. Feature Point Detection and Extraction

The inertial navigation and positioning system can independently perform positioning without relying on other signal sources, and the accuracy in a short time is very high. Therefore, the pedestrian trajectory in a short time can reflect part of the information of the interior building map. With the aid of these characteristics of inertial navigation, the experiment was designed without indoor map information, and the pedestrian’s straight walking phase was detected from the pedestrian path output by short-time inertial navigation. The point of intersection between the adjacent lines is the feature point required by this study.

**Figure 2:**
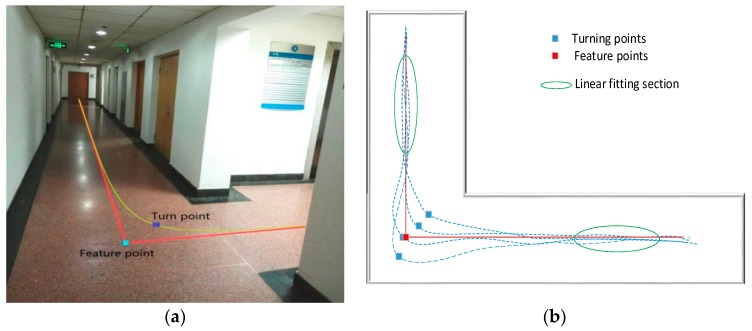
(**a**) Part of the experiment site and (**b**) the schematic diagram of the fitting of the straight walking phase.

In order to clarify the difference and connection between the feature points in this article and the turning points of the pedestrians’ walking process and describe the advantages of correcting pedestrian positions by extracting such feature points instead of pedestrian turning points as benchmarks, the above figure is provided. Figure 2a shows a part of the experiment site. The yellow arc in the figure is the more common turning trajectory of a pedestrian passing through the intersection. Other possible trajectories are shown in blue dotted lines in Figure 2b, where one of the biggest radian parts as defined as the turning point of pedestrian, is shown as the blue square. From the position of the blue square, it can be seen that the position of the pedestrian passing through the corner has randomness. Combined with the actual pedestrian walking characteristics, pedestrians walking in the normal straight-line walking phase are relatively regular. Therefore, the straight-line fitting of pedestrians can be performed by extracting the pedestrians’ straight-line walking phases, as shown in the green ellipse circled in Figure 2b. The extension of the line segment is extended to one point, as shown in the red square in Figure 2b. This point is the feature point to be detected and extracted in this paper. From Figure 2b, it can be seen that such feature points are more suitable for the correction of pedestrian position than the pedestrian turning point. A number of feature points are fitted to a plurality of pedestrian trajectories that pass through the intersection from the same starting point to obtain a plurality of feature points. This position can be seen as the intersection of the interior path of the building. This explanation is more conducive to the understanding of building the feature point map below.

The heading angle is the most important angle information in the attitude angle information. The change of heading angle information was directly used in [12,20] to detect pedestrian-related walking status. However, during the experiment, the sensor was fixed on the waist of the experimenter, and the heading angle information could not occur due to large fluctuations during the straight walking phase. The experiments in this paper placed the IMU sensor fixed on the foot, since pedestrians cannot always ensure that the toe is always parallel to the walking direction even when walking in a straight line, so the heading angle information output by the sensor will appear as larger fluctuations even in the pedestrian walking phase, which can lead to false detections, and therefore it is impossible to observe this effectively in a straight line.

The MEMS-based PDR positioning method is based on the physiological characteristics of the gait of pedestrians. It uses the accelerometer to measure the number of walking steps and the estimated step length and combines the heading information obtained from gyro and magnetic sensors [21]. Using Equation (1) to calculate the current position of the pedestrian.

(1) $$\{\begin{array}{l} X_{t}=X_{t-1}+L_{t}\cos\alpha_{t}\\ Y_{t}=Y_{t-1}+L_{t}\sin\alpha_{t} \end{array}$$

where $\left(X_{t},Y_{t}\right)$ are the coordinates of the position of the pedestrian at time t; $L_{t}$ is the pace of the pedestrian at the time *t*; and $\alpha_{t}$ is the difference of the heading angle of the pedestrian at the moment t.

The pedestrian position calculated by the inertial navigation system based on pedestrian dead reckoning is gradually calculated based on the pedestrian’s position at the last moment, as well as the step and the difference of the heading angle at that moment. Therefore, when a pedestrian walks in a straight line, even if the heading angle fluctuates at a certain moment resulting in a deviation of the pedestrian position, for a period of time, the trajectory of the pedestrian in the straight-line walking stage still exhibits a linear fitting characteristic.

**Figure 3:**
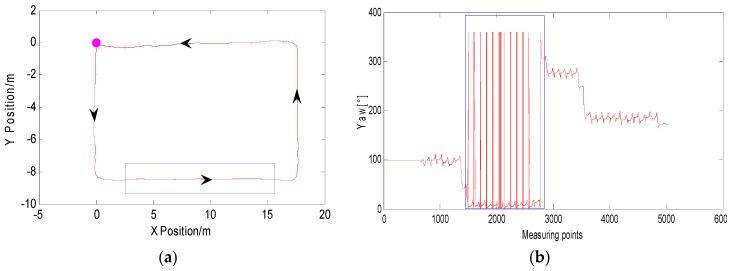
(**a**) Pedestrian trajectory of inertial navigation system (INS) output and (**b**) corresponding heading angle data during walking.

In order to explain this problem more clearly, the following uses a test experiment to illustrate the process of feature point detection and extraction. Pedestrians begin at the starting point and walk counterclockwise along the main indoor path. The walking trajectory is rectangular. As shown in Figure 3a below, the purple dot is the starting point, and the direction of the arrow is the direction of the pedestrian. Due to the short distance and short time, the inertial navigation output position information is more accurate. Figure 3b is the heading angle information diagram of the corresponding IMU output during pedestrian walking. The blue rectangle in Figure 3 is the corresponding pedestrian trajectory information and heading angle data information. It can be seen that the heading angle data will still show fluctuations even if the pedestrian is in a straight-line walking phase. It is not possible to identify effectively whether the pedestrian is in the straight-line walking phase, but the pedestrian trajectory derived from the pedestrian’s dead-reckoning still present linear fitting characteristics.

In order to extract the straight-line walking phase with better linearity from the position data corresponding to the pedestrian trajectory derived from the pedestrian dead-reckoning, a feature point extraction method based on position information was proposed. In the experiment, the mean of all sampling points in 1 s was taken according to Equation (2). On the one hand, it avoids the error of some of the sampling points and avoids the impact of feature detection; on the other hand, the difference of the adjacent data is increased, which is easy to observe and be further treated. The sampling frequency was set to 100 Hz in this article’s experiments.

(2) $$X_{m}=\frac{\sum_{i=1}^{f}X_{i}}{f}\left(t\right),Y_{m}=\frac{\sum_{i=1}^{f}Y_{i}}{f}\left(t\right)$$

In the formula, $X_{m}$ is the mean value of the abscissa of all sampling points in 1 s at time t. $X_{i}$ is the abscissa of all sampling points in 1 s. $Y_{m}$ is the mean value of the ordinate of all sampling points in 1 s at time t. $Y_{i}$ is the abscissa of all sampling points in 1 s. f is the sampling frequency.

**Figure 4:**
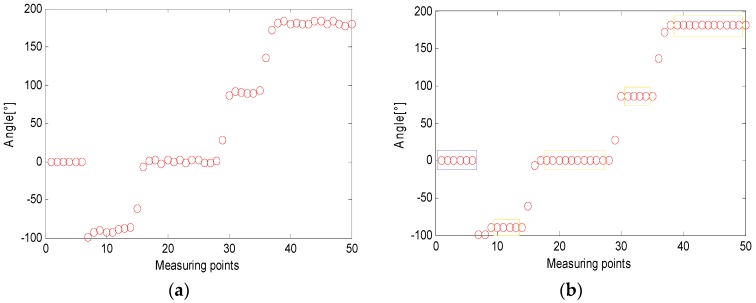
(**a**) Unsmoothed angle information and (**b**) smoothed angle information.

Afterwards, the adjacent average sampling point is calculated according to Equation (3) to obtain the connection angle, and Figure 4a is obtained. After the data is smoothed to obtain Figure 4b, the pedestrian has a static interval before starting to walk to measure the drift of the gyro’s constant value. Therefore, the first segment in the figure is the drift measurement phase and is not included in the calculation as shown by the part inside the blue rectangle in Figure 4b. It can be clearly seen from the figure that the four-segment straight-line walking phase, in order to obtain a better fitting effect, took out the mid-section data of the pedestrian straight-line walking phase and performed straight-line fitting as shown by the part inside the yellow rectangle.

(3) $$\left\{ \begin{array}{c} \theta^{i}=\arctan\left(\frac{Y_{m}^{i}-Y_{m}^{i-1}}{X_{m}^{i}-X_{m}^{i-1}}\right)\\ -\pi <\theta^{i}\leq \pi \end{array}\right.$$

In the formula, $\left(X_{m}^{i},Y_{m}^{i}\right)$ and $\left(X_{m}^{i-1},Y_{m}^{i-1}\right)$ are the coordinates of the adjacent two mean sampling points; and $\theta^{i}$ is the connecting angle of the adjacent mean sampling points.

Use the first-order line fitting of the straight-line walking phase extracted by Equation (4) and obtain the fitted straight line:

(4) $$\{\begin{array}{c} \hat{b}=\frac{\sum_{i=1}^{n}X_{i}Y_{i}-n\bar{X}\bar{Y}}{\sum_{i=1}^{n}X_{i}^{2}-n{\bar{X}}^{2}}\\ \begin{array}{l} \hat{a}=\bar{Y}-\hat{b}\bar{X}\\ p=\hat{a}+\hat{b}q \end{array} \end{array}$$

where $\left(X_{i},Y_{i}\right)$ are the coordinates of the mean position point for the extracted fitting part; $\left(\bar{X},\bar{Y}\right)$ is the mean coordinate of all sampling points representing the fitable parts extracted; and n is the total number of sampling points of the fitable part extracted. $\hat{b}$ is the coefficient of the one-time term for the fitted straight line. $\hat{a}$ is the coefficient of the linear constant term; and p is the straight-line expression.

**Figure 5:**
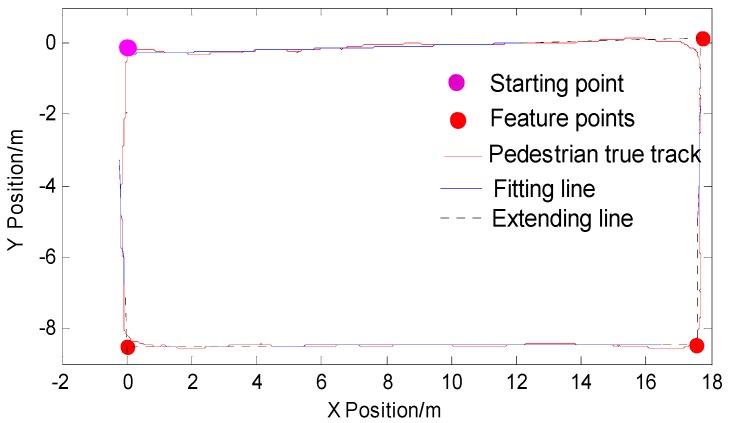
Feature points extraction based on location information.

The feature points extraction method based on the location information is shown in Figure 5. The blue line segment is the straight line fitted by the pedestrians in the straight-line walking phase. The fitting of the adjacent straight-line extension line to the point is the one required for this study. The feature points are shown by the red circle in the figure. In order to obtain more accurate feature points, it was necessary to design the experiment to take multiple values for the same feature point and obtain the average value as the final feature point data in order to construct a more accurate feature point map.

### 3.2. Build the Main Path Feature Point Map

Most of the interior buildings are made up of corridors and rooms. In this paper, we call the corridor paths the main indoor paths, which reflects the general situation of the interior of a building. The feature points on the main path are easier to observe and extract. The variability of the layout of the room is too large and feature point extraction is difficult. Therefore, the feature point extraction and the construction and updating of the feature point map proposed in this paper were aimed at the pedestrian turning point that occurs on the main path.

**Figure 6:**
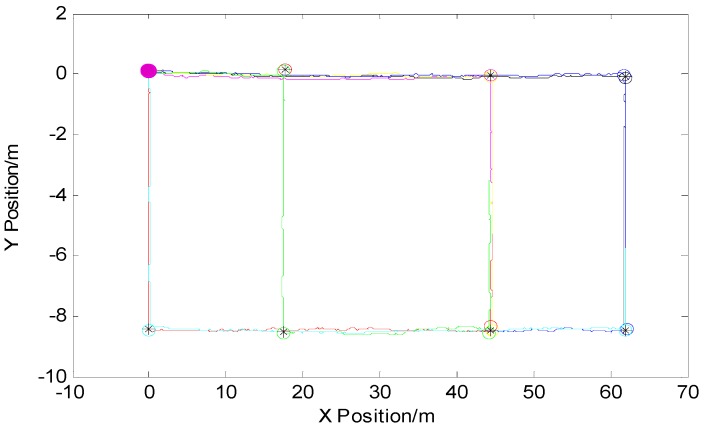
Feature points map.

Based on the short-time high precision of the inertial navigation system and that the pedestrian has the advantages of the autonomy to design experiments, that is, pedestrians have autonomous obstacle avoidance, autonomously select roads that they can travel, and can independently design route-walking characteristics. Each time the experimenter started from the same starting point and walked along the main path of the indoor building, the experiment showed that the shorter the walking time, the fewer the number of turns, and the higher the precision of the INS output trajectory. The trajectory of the same color in Figure 6 was an experimental path, and feature points were extracted according to a feature point extraction method based on position information and are represented by circles of the same color. When designing the experimental path, it was ensured that the intersection of each path passed at least twice, and finally the average value was taken as the required feature point. As each path started from the same starting point, such as the purple dots in Figure 6, the paths were put together and combined with the extracted feature points to build a feature point map that could reflect the main path in the building.

**Figure 7:**
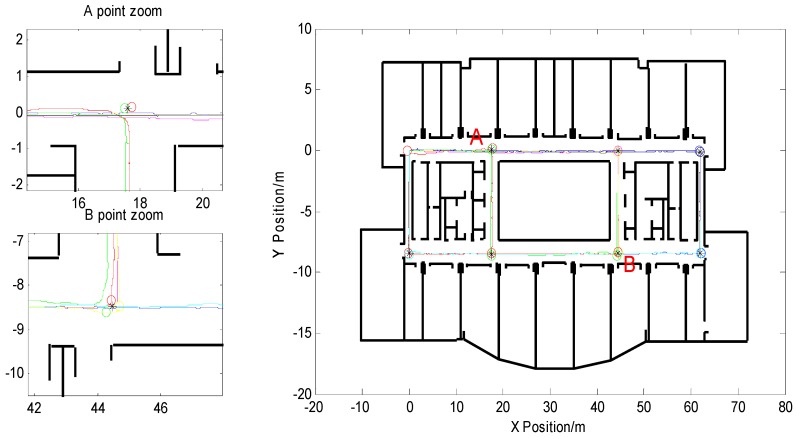
Matching of feature point maps with interior building plans and the details of the two zoomed-in feature points.

In order to verify the accuracy of the feature point map, the maps were matched with the experimentally selected indoor architectural map. The matching result is shown in Figure 7. From the figure, it can be seen that the extracted feature points can be seen as a cross point of the route within the allowable error range, and the constructed feature point map can reflect the approximate path of the interior building. The details of the two zoomed-in feature points are as shown in the left side of the picture corresponding to points A and B, respectively. At point A, a pedestrian passes through the intersection twice. Each time the extracted feature points are shown in the different color circles, the average of the two feature points is output to obtain the feature points required by this article, as shown in black “※” in the Figure 7. Point B is the same as point A: the pedestrian passes the intersection three times.

The feature point map not only contains the extracted feature point information, but also includes the position information of each point on the main path, thereby distinguishing whether the pedestrian turning point occurs on the main path or inside the house, which provides ideas for the following new feature point extraction. That is, this position information constitutes the main path database, and the extracted new turning point is matched with the main path database one by one. If the matching distance is less than a given threshold, it is determined that the turning point occurs on the main path. Judging by Equation (5):

(5) $$\left\{ \begin{array}{c} {\left(d_{T}^{n}\right)}^{i}=\sqrt{{\left(x_{T}^{n}-x_{l}^{i}\right)}^{2}+{\left(y_{T}^{n}-y_{l}^{i}\right)}^{2}}\\ \ell_{T}^{n}=\{\begin{array}{cc} 1 & {\left(d_{T}^{n}\right)}^{i}\leq Th\\ 0 & others \end{array} \end{array}\right.$$

In the formula, $\left(x_{T}^{n},y_{T}^{n}\right)$ is the coordinate of the nth turn point extracted; $\left(x_{l}^{i},y_{l}^{i}\right)$ is the coordinate of the ith position of the main path database; and ${\left(d_{T}^{n}\right)}^{i}$ is the distance between the extracted nth turn point and the ith position point of the main path database. Th is the threshold value, which is determined for the turning point state, and the specific value needs to be determined according to the actual situation of the experimental site. $\ell_{T}^{n}=1$ indicates the extracted nth turn point that occurs on the main path. $\ell_{T}^{n}=0$ indicates that the extracted nth turn point is not on the main path.

## 4. Design of the Particle Filter-Simultaneous Localization and Map Building (PF-SLAM) Indoor Pedestrian Positioning System Based on Feature Point Map

### 4.1. SLAM Problem Description

Simultaneous localization and mapping, abbreviated as SLAM, means that when a moving object is completely unfamiliar to its environment, its location is not determined, such as emergency rescue missions, for example, fire rescue. In this case, by creating a surrounding environment map and then using the created environment map to help the user to achieve their own positioning, SLAM mainly solves two major problems. These two problems are mutually premised and interact with each other: first, an environment map is constructed. This is achieved by collecting information about the surrounding environment through various sensors worn on the carrier. However, this information is often large and unorganized, and feature information needs to be extracted from this information and aggregated into an environment map. Second, the user’s positioning determines the location of the user in the constructed environment map.

The SLAM idea is the core idea of the entire positioning system. The main implementation steps of the SLAM algorithm can be summarized in the following steps:

(1) Feature points are extracted, and the main path feature point map is constructed. The INS system obtains the information of the pedestrian location, the accuracy of which is determined by the accuracy of the sensors and the sampling time. The feature points of the indoor main path are constructed by extracting the feature points from the straight-line fitting of the straight pedestrian walking phase through the more accurate main path attitude information obtained.
(2) Location prediction. The heading angle difference is corrected in the detected pedestrian straight-line walk state, thereby suppressing the accumulation of the heading angle error. The pedestrian’s position and posture are predicted by the corrected heading and particle filter algorithm based on pedestrian dead reckoning.
(3) Feature point matching. Determine whether the observed pedestrian position is a turning point. If yes, match the feature point and update the particle weight and particle distribution. If it cannot be matched with the current feature point database, the inflection point on the main path of the pedestrian is extracted as a new feature point and added to the feature point map.
(4) Correction. The pedestrian position is corrected according to the feature point matching result, and the detected turning point on the main path, which when unmatched is used to expand and update the feature point map information.

### 4.2. PF-SLAM System Model

The SLAM system in a practical project is a non-linear system where the noise does not follow the Gaussian distribution. If the method based on the extended Kalman filter-SLAM (EKF-SLAM) is used to estimate the non-linear non-Gaussian state, linear errors will inevitably be introduced into the system, and particle filtering based on Monte Carlo and sequential importance sampling (SIS) is suitable for processing non-linear non-Gaussian systems. It is an effective method for processing SLAM systems. Therefore, the SLAM method based on particle filtering was used in this paper.

Particle filtering is implemented by the non-parametric Monte Carlo simulation method for Bayesian filtering. The prior and a posteriori information is described in sample form instead of function. The SLAM problem needs to be solved by calculating the joint posterior probability density distribution of the pedestrian pose and feature point map:

(6) $$P(x_{1:k},\theta |z_{1:k},u_{1:k})$$

In the formula, $x_{1:k}={\left[x_{1},x_{2},\cdots ,x_{k}\right]}^{T}$ represents the pedestrian walking path up to time k; $\theta ={\left[x_{1},y_{1},x_{2},y_{2},\cdots ,x_{n},y_{n}\right]}^{T}$ indicates the position of the feature point in the feature point map; $z_{k}$ is the observation, note $z_{1:k}={\left[z_{1},z_{2},\cdots ,z_{k}\right]}^{T}$ represents the observation sequence up to time k; $u_{k}$ is the control input, note $u_{1:k}={\left[u_{1},u_{2},\cdots ,u_{k}\right]}^{T}$ represents the control input sequence up to time k.

The core idea of particle filtering SLAM is to factorize the joint posterior probability distribution into the pedestrian path part and the map part with the pedestrian path as the condition.

(7) $$P(x_{1:k},\theta |z_{1:k},u_{1:k})=P(\theta |x_{1:k},z_{1:k},u_{1:k})P(x_{1:k}|z_{1:k},u_{1:k})$$

If the pedestrian’s entire path $x_{1:k}$ is given, the map θ can be calculated analytically. Therefore, the posterior probability of the pedestrian’s pose $P(x_{1:k}|z_{1:k},u_{1:k})$ can be estimated by the particle filtering method. Based on this, the map θ can be recursively calculated by the Kalman filter.

The particle importance weights are:

(8) $$w_{k}^{\left(i\right)}\propto P(z_{k}|x_{k}^{\left(i\right)})w_{k-1}^{\left(i\right)}$$

where $w_{k}^{\left(i\right)}$ is the weight of the particle at the moment;$w_{k-1}^{\left(i\right)}$ is the weight of the particle at the previous moment; and $P(z_{k}|x_{k}{}^{\left(i\right)})$ is the posterior probability of pedestrian position.

Perform particle weight normalization:

(9) $$w_{k}^{\left(i\right)}=\frac{w_{k}^{\left(i\right)}}{\sum_{i=1}^{N}w_{k}^{\left(i\right)}}$$

where N is the total number of particles.

Doucet [22] showed that the variance of weights increases with time, which leads to the degeneracy problem that prevails in particle filtering. An appropriate measure of degradation is the adoption of effective populations $N_{eff}$. The smaller $N_{eff}$ indicates that the degradation is more serious. $N_{eff}$ can be calculated by the following equation [12]:

(10) $$N_{eff}=\frac{1}{\sum_{i=1}^{N}{\left(w_{k}^{\left(i\right)}\right)}^{2}}$$

The re-sampling method is often used to solve the degeneracy phenomenon, and re-sampling is performed when the number of effective particles is less than a given number. Given a valid particle threshold $N_{threshold}$, resampling is performed when the number of effective particles $N_{eff}\leq N_{threshold}$, thereby maintaining a consistency algorithm.

### 4.3. Design of the PF-SLAM Localization Algorithm Based on Feature Point Map

**Figure 8:**
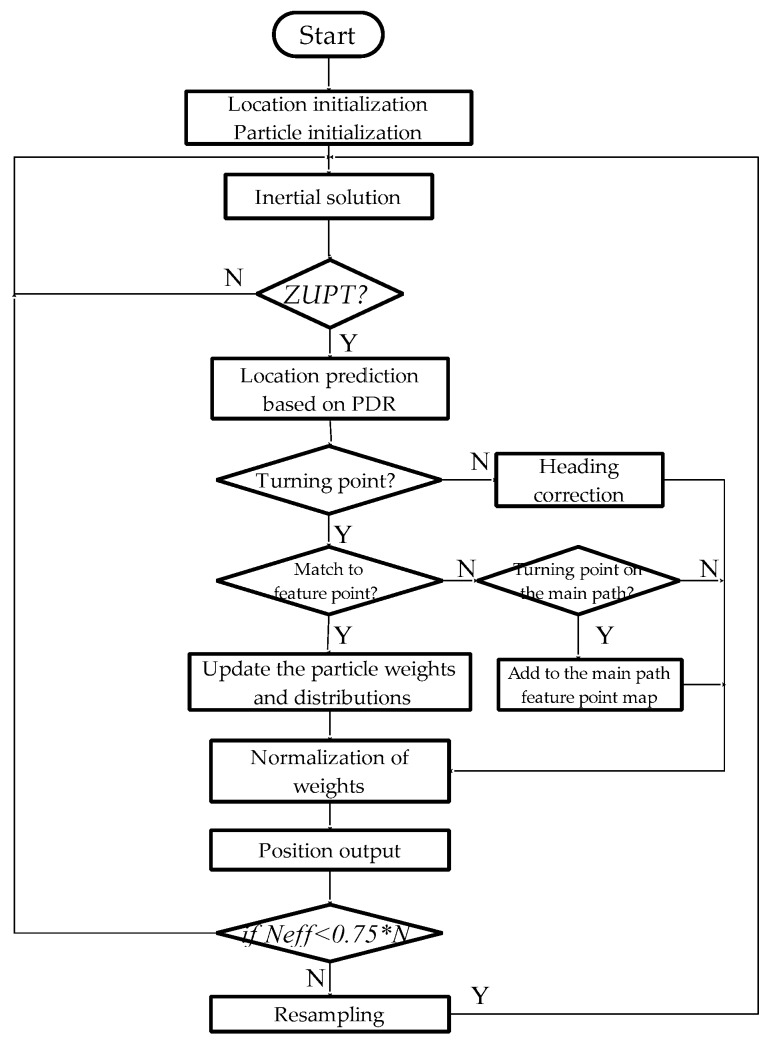
Flow chart of the particle filter-simultaneous localization and map building (PF-SLAM) location algorithm based on the feature point map.

Figure 8 is a flow chart of the PF-SLAM location algorithm based on the feature point map. Accelerometer and gyro output data are used for inertial navigation, and the step and heading information for each step of the pedestrian is obtained through the ZUPT algorithm. Particle filtering based on dead reckoning predicts the pedestrian position. The state of pedestrians at the moment is judged by the turn-straight-state threshold detection method. If it is a straight walking stage, the heading angle difference is corrected, and the heading angle error accumulation is suppressed. If it is a turning point, then feature point matching is performed. If it matches the feature point stored in the current feature point map, then the particle is updated as the feature point position and its weight based on the position relationship of all particles and feature points at the moment is updated, and the weight is normalized and the pedestrian position output. If the turning point cannot match the existing feature point database, it does not update the weight of the particle, and it is judged as to whether it belongs to the turning point on the main path. If yes, the pedestrian position data are added to the feature point database of the feature point map to update the feature point map. Finally, it is judged as to whether the particles need to be re-sampled. Such a loop realizes the positioning of indoor pedestrians.

In summary, the main modules of the system include an inertial navigation calculation module based on ZUPT, a main path feature point map module, and a particle correlation-based data association module. The inertial navigation calculation module based on ZUPT provides motion information to the system; the main path feature point map module provides the system with real-time observed feature point information and saves the real-time updated feature point map information; the data correlation module based on the particle filter is the core of the whole system. It mainly suppresses the accumulation of the heading angle error according to the observed values of the system and performs data association processing with the feature point map and updates the weight of the particle and obtains the final matching result. Therefore, we will focus on the introduction of how to suppress the accumulation of the heading angle errors based on particle filtering and how to perform feature point matching and particle weight update.

#### 4.3.1. The Turn-Straight-State Threshold Detection Method

In the actual project, although the foot-tied inertial navigation system can introduce the zero-speed observation when the foot touches the ground through the ZUPT stage, and then update the principle according to the Kalman filter, the acceleration drift error is reduced. However, for gyro errors, there are no reliable observations, leading to serious drift in long-distance heading errors. Therefore, this paper proposed the turn-straight-state threshold detection method to suppress the heading error accumulation over time.

The particle filter algorithm based on pedestrian dead reckoning will output one-step heading angle information at every pedestrian zero speed point. Pedestrian heading angle information can reflect the pedestrian movement state to some extent. Although the heading angle change model is inaccurate, there is significant variation in the difference of the heading angle between the pedestrian turning stage and the straight walking stage. Therefore, this paper proposed a turn-straight-state threshold detection method to determine the pedestrian status. Pedestrian turning points were matched with the feature point maps to perform position correction and particle weight updating. Pedestrians were detected in the straight-line walking phase and the heading angle difference was corrected to suppress the accumulation of heading angle errors. The difference of the heading angle is:

(11) $$\vartheta_{n}=yaw_{n}-yaw_{n-1,}\;n=2,3\cdots ,m$$

where $\vartheta_{n}$ is the difference of the heading angle of the nth zero velocity point; $yaw_{n}$ is the heading data of the nth zero velocity point; $yaw_{n-1}$ is the heading data of the n−1th zero velocity point; $\vartheta_{n}$ is the initial heading when n=1; and m is the total number of steps for pedestrians during the experiment.

Combined with the actual situation, when the pedestrian walks in a straight line, the heading angle value remains basically unchanged, so the heading angle difference was set to 0 to suppress the accumulation of the heading angle error. Due to the pedestrian’s discretion when turning the corner, and the corner angle of the building actually being different, the corresponding heading angle difference will be different and will only detect the turning phase without modifying the value. The corresponding setting of the turn-straight-state threshold detection method is as follows:

(12) $$\theta_{n}=\{\begin{array}{ll} \vartheta_{n} & Th1<|\vartheta_{n}|<Th2\\ 0 & others \end{array}$$

where $\theta_{n}$ is the difference of the corrected heading angle of the nth zero velocity point; $\vartheta_{n}$ is the difference of the before correction heading angle of the nth zero velocity point; Th1, Th2 are the state judgment threshold where the specific value needs to be determined according to the accuracy of the IMU and the specific conditions of the experiment. 

#### 4.3.2. Feature Point Matching

Although the turn-straight-state threshold detection method can effectively suppress the pedestrian heading error accumulation as a pedestrian walks straight, the particle filter derived from the Monte Carlo and recursive Bayesian estimation has random characteristics. Even though the heading information is corrected, it is inevitable that there will be a serious divergence of particles at the inflection point, which will result in positioning failure. For this reason, with the aim of addressing the problem where the inaccuracy of the heading angle error model in the particle filtering equations causes the particles to be severely divergent at the corners, a feature point matching algorithm was proposed. Corresponding feature point matching through the detected pedestrian turning point and feature point map and the pedestrian turning point were matched to the relatively accurate feature point and the particle weight was updated to ensure the effective and correct propagation of the particle.

**Figure 9:**
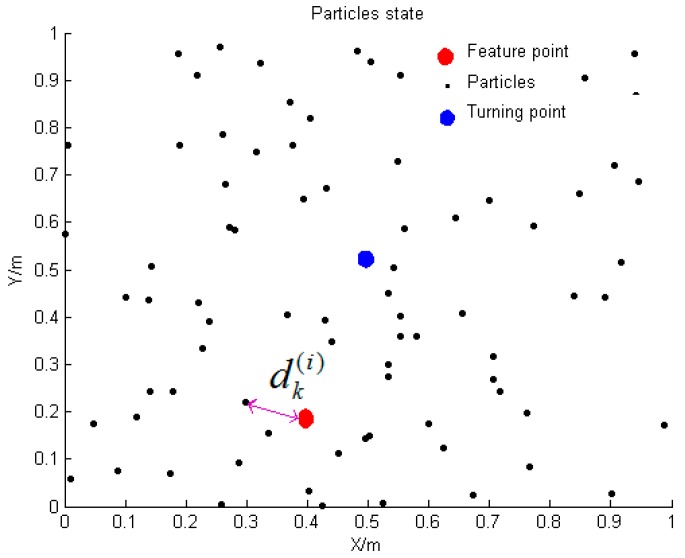
Feature point matching diagram.

As shown in Figure 9, the black particles in the figure are partial particles at a certain moment. The blue dots in the figure are the positions of the pedestrians obtained by weighting all particle positions at that moment. If it is detected that the pedestrian is at the turning point at this moment, the output position of the particle at this moment is updated to the position of the matched characteristic point, that is, at the red circle in the figure. The particle filter makes the weight information of the particles limited by the feature point information after being integrated into the feature point map [23]. After the feature point matching is successful, the particle weight is updated according to the distance between each particle position and the matched feature point at the moment, and the updated weight is assigned to the next moment, so the particles continue to propagate forward. The particle weights are updated as follows:

(13) $$w_{k}^{\left(i\right)}=\{\begin{array}{cc} w_{k-1}^{\left(i\right)} & unmatched\\ P(z_{k}|x_{k}{}^{\left(i\right)})w_{k-1}^{\left(i\right)} & matched \end{array}$$

$P(z_{k}|x_{k}{}^{\left(i\right)})$ is the posterior probability of pedestrian position and this article can be expressed as:

(14) $$P(z_{k}|x_{k}{}^{\left(i\right)})=\frac{1}{\sqrt{2\pi }}e^{-\frac{{\left(d_{k}^{\left(i\right)}\right)}^{2}}{2}}$$

where $d_{k}^{\left(i\right)}=\sqrt{{\left(x_{k}^{\left(i\right)}-x_{T}\right)}^{2}+{\left(y_{k}^{\left(i\right)}-y_{T}\right)}^{2}}$ indicates the distance between the ith particle and the matched feature point at time k.

## 5. System Experiment and Result Analysis

**Figure 10:**
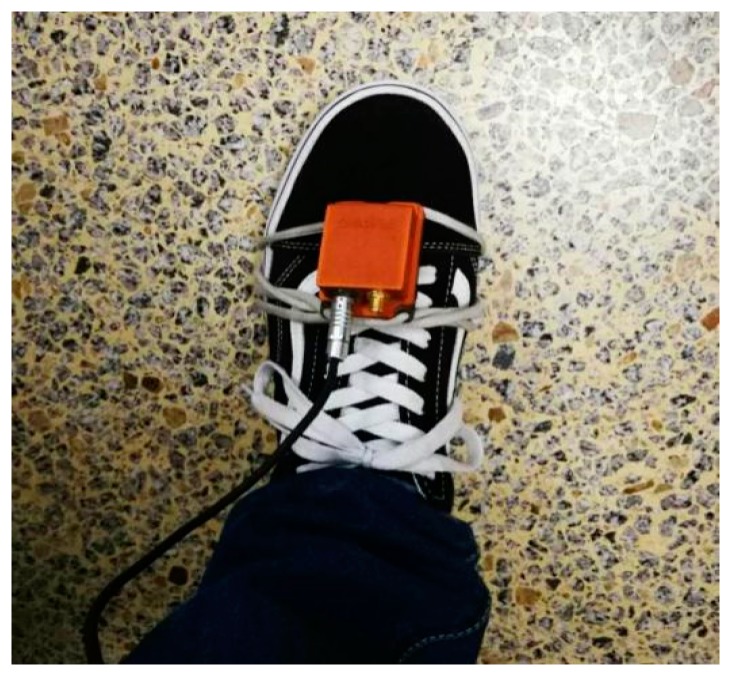
Inertial measurement unit (IMU) fixed to the experimenter’s foot.

The system does not rely on other equipment, only relying on the IMU to collect data; the navigation computer receives the IMU sampled data through the USB interface and can be stored in real time. The navigation computer adopts an ordinary PC to implement the inertial navigation solution, filtering algorithm, building feature point maps, feature point matching and other algorithm steps, and ultimately achieves pedestrian indoor positioning. The number of particle filtered particles in the experiment was 500. The IMU consists of a three-axis MEMS gyroscope and a three-axis MEMS accelerometer. The inertial measurement device IMU is fixed to the foot of the pedestrian, as shown in Figure 10.

**Figure 11:**
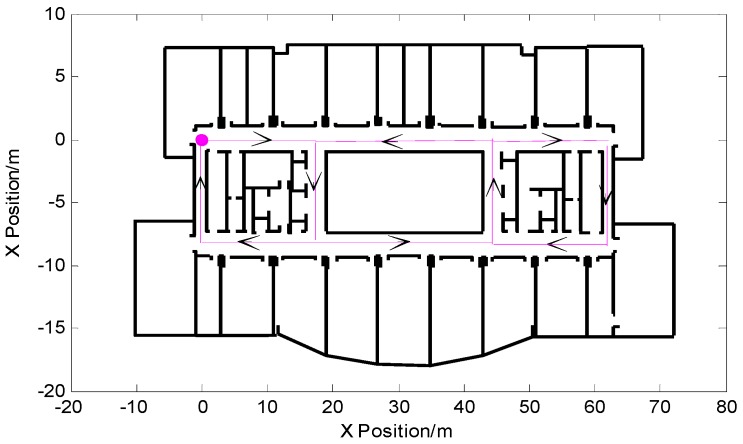
Experimental site and preset walking path.

In order to verify the proposed PF-SLAM indoor pedestrian location algorithm based on feature point map proposed in this paper, the experimental site selected for this study was the tenth floor of the science building of Beijing University of Technology. The experimental area was 80 × 25 square meters. The concrete building information map and the experimental ideal route are shown in Figure 11. The purple circle in the figure shows the starting point of the pedestrian and was also the starting point of the path of the main path feature point map, thus avoiding the experimental error caused by the inaccuracy of the initial positioning. The purple trajectory in Figure 11 is the experimental preset pedestrian trajectory. The experimenter started with (0,0) and eventually returned to the starting point. The direction of the black arrow is the walking direction of the pedestrian, and the overall path shows a closed twisted shape.

**Figure 12:**
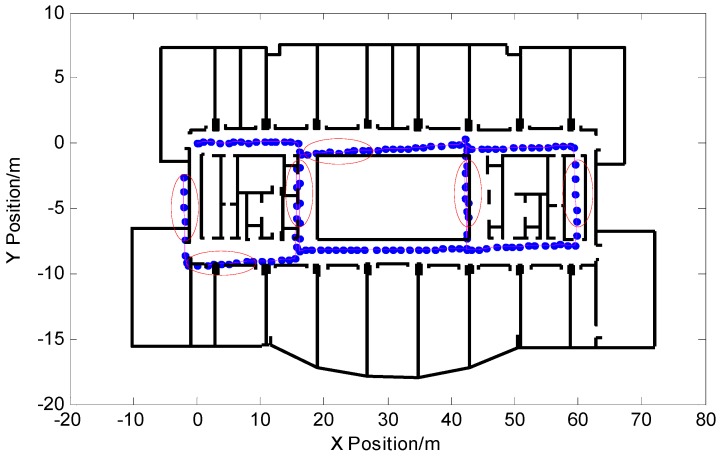
Pedestrian trajectory output by particle filter based on pedestrian dead reckoning.

In order to see the effect of the algorithm more clearly, the pedestrian trajectory based on the pedestrian dead-reckoned particle filter output was combined with the indoor building plan. Figure 12 shows the results of the particle filter output without adding the proposed algorithm. The experimental walking time was 176.65 s, a total of 142 steps, and walking distance was 184.60 m. The pedestrian’s final position coordinate was (2.039,2.646). From the part enclosed by the red ellipse in the figure, it can be seen that the pedestrian trajectory of the output showed the serious phenomenon of passing through the wall, and the positioning of some locations failed.

**Figure 13:**
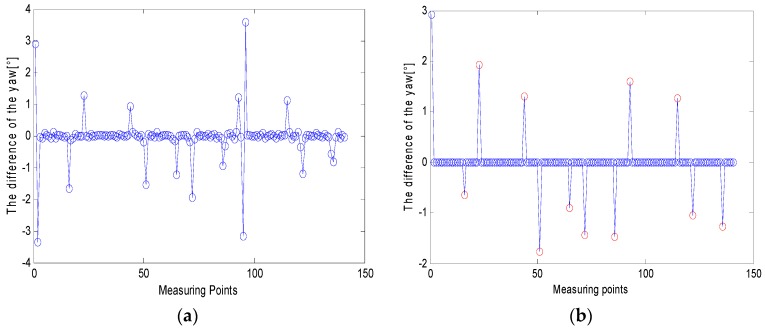
(**a**) The difference of the heading angle before corrected and (**b**) the difference of the heading angle after correction.

Figure 13a is the difference of the heading angle corresponding to each zero velocity point of the pedestrian in the above experiment. From the figure, it can be seen that the difference of the heading angle cannot effectively observe the state of the pedestrian. Through the turn-straight-state threshold detection method proposed in this paper, the turning point and the pedestrian walking straight phase were separately analyzed; the pedestrian’s turning point (marked in red) can be detected as shown in Figure 13b, and at the same time, as the detected pedestrians walked in a straight line, the difference of the heading angle was set to zero to suppress heading error accumulation. According to Equation (12) and the IMU’s accuracy and experiment conditions, the threshold was set to:

(15) $$\theta_{n}=\{\begin{array}{cc} \vartheta_{n} & 0.9<|\vartheta_{n}|<1.9\\ 0 & others \end{array}$$

**Figure 14:**
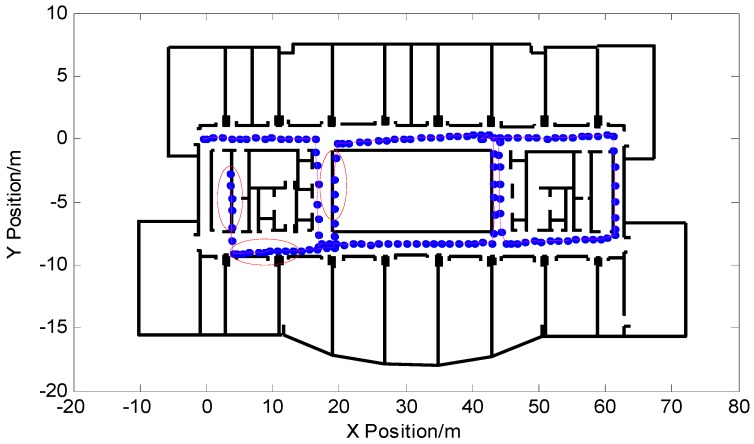
Correction of pedestrian trajectory based on the turn-straight-state threshold detection method.

The pedestrian trajectory corrected by the turn-straight-state threshold detection method proposed in this paper is shown in Figure 14. It can be seen from the figure that the output of the pedestrian trajectory through the wall was reduced, but as time increased and the number of pedestrian steps increased, there will still be a trajectory through the wall in the later period, resulting in positioning failure.

**Figure 15:**
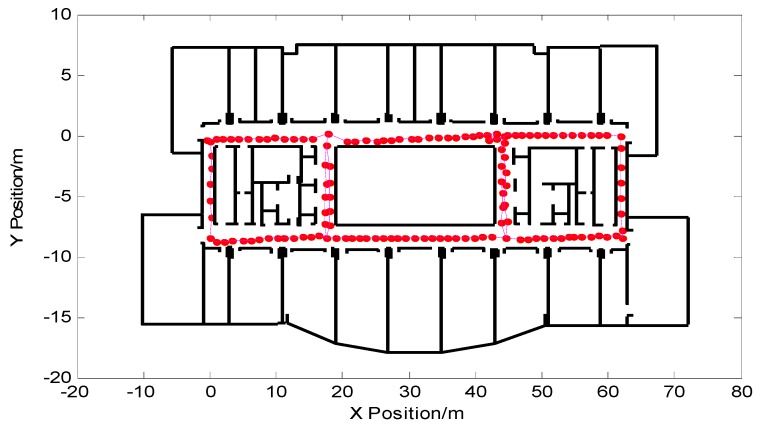
Pedestrian interior trajectory corrected based on this algorithm.

The detection of the pedestrian turning point was matched with the feature points in the feature point map, and the pedestrian turning point position was reset to the matched feature point position, and the weight value was updated at the same time, while the position drift was modified to reduce the degree of divergence of the particle at the corner. The corrected pedestrian trajectory is shown in the red trajectory of Figure 15. The pedestrian’s final position coordinate was (0.1138,−0.4618) after correction. From the figure, it can be seen that the PF-SLAM algorithm based on the feature point map proposed in this paper effectively suppressed the accumulation of the heading error of the particle filter over time. Compared with the pedestrian trajectory before correction, the phenomenon of passing through the wall of particles was basically solved, and the positioning accuracy of indoor pedestrians was greatly improved.

**Figure 16:**
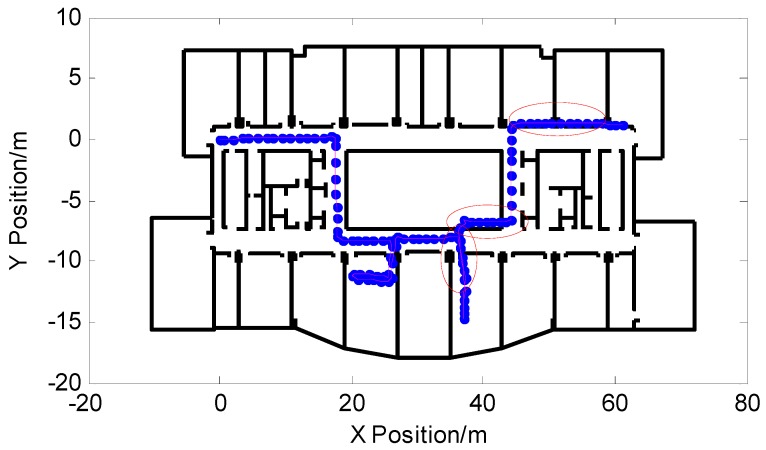
Pedestrian trajectory before correction.

The turning points of the walking process of the above experimental pedestrians all matched the stored feature points in the feature point map. In the following experiments, pedestrians still started from the same starting point and entered two rooms. The experimenter started with (0,0) and eventually returned to the starting point. The experimenter started with (0,0) and stopped at the preset final position point (62,0). The experimental walking time is 139.46 s, a total of 107 steps, and walking distance is 118.74 m. Figure 16 shows the pedestrian trajectory of the particle filter output before correction. The pedestrian’s final position coordinate was (61.04,1.236) before correction. It can be seen from the figure that the particles appeared through the wall and some of the trajectory positioning failed.

**Figure 17:**
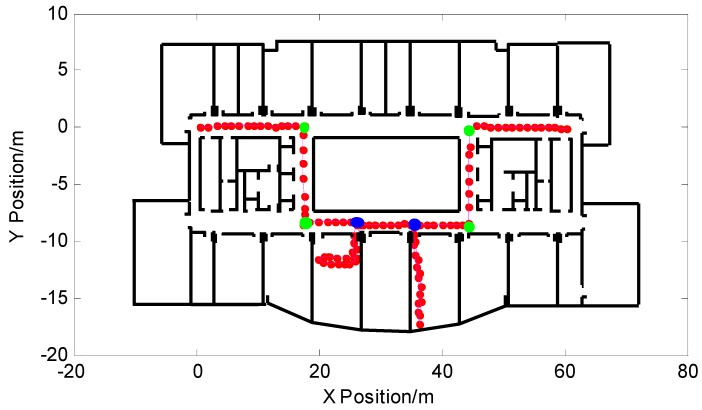
Pedestrian trajectory after correction.

The PF-SLAM indoor pedestrian positioning algorithm based on feature point map proposed in this paper corrected the pedestrian heading and corrected the pedestrian position error through feature point matching. The corrected pedestrian trajectory is shown in Figure 17, according to Equation (5) and the width of the experimental site corridor, the turning point status was determined as:

(16) $$\ell_{T}^{n}=\{\begin{array}{cc} 1 & {\left(d_{T}^{n}\right)}^{i}\leq 0.5\\ 0 & others \end{array}$$

In Figure 17, the trajectory basically solved the phenomenon of particles through the wall, and the positioning accuracy was greatly improved. The pedestrian’s final position coordinate was (61.47,−0.0623) after correction. The green circles in the figure represent the turning points that were matched to the feature points. The remaining turning points represent the pedestrian turning points that were not matched to the current feature point database and extracted by the turn-straight-state threshold detection method. According to the stored main path position data information in the feature point map, only the turning point on the main path was extracted and added to the feature point map to update the map, as shown by the blue circle.

In order to further evaluate the accuracy of the indoor pedestrian positioning algorithm proposed in this paper, Table 1 shows a comparison of the parameters before and after the correction of the algorithm. The position error is calculated by dividing the distance between the preset final position and the experimental output final position by the percentage of the total distance. It can be seen from Table 1 that the PF-SLAM indoor pedestrian location algorithm based on a feature point map algorithm proposed in this paper improves the indoor pedestrian positioning accuracy and greatly reduces the number of positioning efforts that failed due to particles passing through the wall.

## 6. Conclusions

This paper studied the indoor pedestrian positioning algorithm and proposed a PF-SLAM indoor pedestrian positioning algorithm based on a feature point map. Without prior knowledge of the map, the indoor pedestrian positioning could be achieved only through the inertial navigation device IMU and provided a solution for the situation of emergency rescue and the inability to obtain indoor maps in a timely manner. The PF-SLAM algorithm was implemented by building a feature point map of the indoor main paths through the extracted feature points, and the main path feature point map can reflect the general situation in the interior of the building. A turn-straight-state threshold detection method was proposed to detect the pedestrian straight-walking phase and turning points. In the straight-walking phase, the difference of the heading angle was corrected to suppress the heading error accumulating over time. The detected turning points of the main path were matched with the feature point map and the weights were updated, effectively suppressing the divergence of the particles at the inflection point. Experimental results showed that the indoor pedestrian positioning algorithm proposed in this paper could effectively suppress the accumulation of heading errors, and the positioning results were more accurate. It effectively solves the problem of positioning failures that rely solely on strapdown inertial navigation.

## Figures and Tables

**Table 1:** Comparison of parameters before and after correction of the algorithm.

	Parameters	Experiment 1	Experiment 2
Before Correction	Position error (%)	1.8	1.3
	Number through wall	6	3
After Correction	Position error (%)	0.26	0.42
	Number through wall	0	0
